# Synthesis and properties of Fe_3_O_4_-activated carbon magnetic nanoparticles for removal of aniline from aqueous solution: equilibrium, kinetic and thermodynamic studies

**DOI:** 10.1186/1735-2746-10-19

**Published:** 2013-02-17

**Authors:** Babak Kakavandi, Ahmad Jonidi, Roshanak Rezaei, Simin Nasseri, Ahmad Ameri, Ali Esrafily

**Affiliations:** 1 Department of Environmental Health Engineering, School of Public Health, Tehran University of Medical Sciences, Tehran, Iran; 2 Faculty of Medical sciences, Faculty of Medical sciences, Tarbiat Modares University, Tehran, Iran; 3 Center for Water Quality Research (CWOR), Intitute for Environmental Research (IER), Tehran, University of Medical Sciences, Tehran, Iran

**Keywords:** Fe_3_O_4_-activated carbon magnetic nanoparticles, Adsorption, Aniline, Kinetics, Thermodynamic

## Abstract

In this study, powder activated carbon (PAC) and magnetic nanoparticles of iron (III) oxide were used for synthesis of Fe_3_O_4_-activated carbon magnetic nanoparticles (AC-Fe_3_O_4_ MNPs) as an adsorbent for the removal of aniline. The characteristics of adsorbent were evaluated by SEM, TEM, XRD and BET. Also, the impact of different parameters such as pH, contact time, adsorbent dosage, aniline initials concentration and solution temperature were studied. The experimental data investigated by Langmuir and Freundlich adsorption isotherms and two models kinetically of pseudo first-order and pseudo second-order. The results indicated that the adsorption followed Langmuir and pseudo second-order models with correlation r^2^ > 0.98 and r^2^ > 0.99, respectively. The equilibrium time was obtained after 5 h. According to Langmuir model, the maximum adsorption capacity was 90.91 mg/g at pH = 6, and 20°C. The thermodynamic parameters indicated that adsorption of aniline on magnetic activated carbon was exothermic and spontaneous. This synthesized AC-Fe_3_O_4_ MNPs due to have advantages such as easy and rapid separation from solution could be applied as an adsorbent effective for removal of pollutants such as aniline from water and wastewater.

## Introduction

Rapid increasing of industries and subsequently increasing the disposal of pollutants especially organic compounds, to the water resources and environment caused serious and adverse environmental impacts. Aniline is a simple matter with aromatic ring that has a benzene ring and a NH_2_ bond. It’s used as raw material in petrochemical and agrochemical industries, industries of production of pesticides, rubber, plastics, pharmaceuticals and dyestuff, as well as by-product from paper and textile industries [1,2]. Aniline was dissolved in water as 3.5% and this solubility increases the presence possibility of this pollutant in such sources. This compound is toxic, carcinogenic and mutagenic, and its presence in human blood caused change of hemoglobin to the Methemoglobin and eventually to disease Cyanosis. Also reiterate and lengthy exposures can cause kidney, Liver, bone and neurological marrow disorders and also loss of anemia, appetite, and weight loss. Thus, aniline presence in water resources even at low concentrations is injurious to aquatic organisms and human health [3,4]. America Environmental Protection Agency (USEPA) and Europe Economic Committee (EEC) have classified aniline as refractory pollutant [5].

According to the chemical characteristics and environmental impacts of aniline and the inefficiency of conventional treatment methods for the complete removal of it from the aquatic solution, applying of advanced, quick and effective techniques, is essential. Mainly the most common processes for aniline removal consists of oxidation with ozone [6], adsorption with activated carbon, clays and other adsorbent [7,8], biodegradation [9], electrolysis [10] and ligand exchanger [11]. But the complete removal of aniline and its derivatives with some of this process is difficult or even impossible and in decomposition processes such as biodegradation and electrolysis, very high cost is the limiting factor. The adsorption process by activated carbon rather than other techniques to remove pollutants even at very low concentrations, because of being in sensitive to pollutants and toxic compounds, allowing recovery of both adsorbent and adsorbate and not forming of dangerous material, such as ozone and free radicals, is very efficient [12,13].

Activated carbon, due to its structure and high surface area, has been proposed as an adsorbent, and a suitable option for the effective removal of organic contaminations (especially hard-biodegradable pollutants) from aquatic environment. But using of it in large scales (in engineering processes) has been limited because of problems such as filtration, dispersion, create turbidity and high cost of its reduction [14].

Recently, magnetic separation method has been widely used due to low cost, simplicity and being quick in separation and high efficiency. In this regard, different absorbents such as ion exchange resins, zeolites, activated carbon fibers, polymeric adsorbent and waste and even nanoparticles have been magnetized [15]. The prerequisite for magnetic separation is the synthesizing or combining them with nanoparticles (metal oxides). These nanoparticles that are mainly in the form of Fe_3_O_4_ MNPs, were separated or removed accompany with the target pollutants from the aquatic environment by a magnet. Furthermore, the presence of magnetic iron oxide (Fe_3_O_4_) leads to chemical stability, low toxicity, and excellent recyclability of adsorbent and these have been caused to widely use of this method for removal of toxic ions and organic contaminants from water and wastewater and according to conducted studies these material itself, have high capacity to remove pollutants [16]. In this study new bed has been provided to aniline removal and laboratory conditions have been optimized to increase the absorption capacity. Therefore, the aim of this study was synthesizing magnetic nanoparticles of Fe_3_O_4_- activated carbon (AC-Fe_3_O_4_ MNPs) as an adsorbent for the adsorption of aniline from synthetic wastewater samples.

## Materials and methods

### Chemicals and instruments

All the components were analytical reagent-grade and used as supplied. Nitric acid (HNO_3_ 65%), ferric nitrate (Fe (NO_3_)_3_.9H_2_O), powder activated carbon (PAC) and aniline were purchased from Merck. In all of experiments is used double distilled deionized water and pH meter (HACH-HQ-USA) for control pH solution (±0.01) and for determination of aniline residual concentrations applied UV-Visible spectrophotometer (CECIL CE7400). In order to magnetic separation of adsorbent from aqueous solution also used a magnetic field with an intensity of 1.3 T (5 × 5 × 4 cm).

### Preparation of activated carbon-Fe_3_O_4_ magnetic nanoparticles (AC-Fe_3_O_4_ MNPs)

The Fe_3_O_4_-activated carbon magnetic nanoparticles (AC-Fe_3_O_4_ MNPs) were prepared by a chemical co-precipitation method. Synthesis of Fe_3_O_4_ nanoparticles and combine it with activated carbon was according to the technique given by Do et al. with small alters [17]. At the first, especially amount of activated carbon was impregnated in the nitric acid (63%) to 3 h in 80°C for make hydrophilic with using an ultrasonic bath. The sample then were filtrated and dried in a room temperature. Subsequently, 5 g of obtained powder impregnated into a 200 mL of aqueous solution containing Fe_3_O_4_.9H_2_O and placed in ultrasonic vibration for a 1 h at 80°C. Then sample was filtrated and dehydrated in an oven at 105°C for 1 h. The samples were heated inside of a furnace in 750°C within 3 h under nitrogen gas in other to formation of AC-Fe_3_O_4_ magnetic nanoparticles. Finally, the synthesized adsorbent two washed with deionized water four times and then dried at a 105°C and kept in desiccators for use.

### Characterization of the adsorbent

The specific surface area, volume and pore size distributions of AC-Fe_3_O_4_ magnetic nanoparticles were measured by Brunner, Emmett and Teller (BET) and Barrett, Joyner and Halenda (BJH)(Quantachrome, NOVA2000) using method of N_2_ isotherms. In order to determination of composition crystalline structures and X-ray diffraction (XRD) pattern nanoparticles Fe_3_O_4_ applied of a powder X-ray diffraction (Quantachrome, NOVA2000) using Cu-kα radiation, λ = 1.54 Å at 25°C. The surface physical morphology, shape and size of Fe_3_O_4_ nanoparticle were analyzed using scanning electron microscopy (SEM, PHILIPS, XL-30) and transmission electron microscopy (TEM, PHILIPS, EM 208).

### Aniline adsorption optimization

At first standard stock solution of aniline at a 1000 mg/L was prepared by dissolving required amount in distilled water. Then stock solution diluted by distilled water to prepare different concentrations. The experiments of aniline adsorption onto AC-Fe_3_O_4_ MNPs carried out by batch adsorption method. All experiments were done on 100 mL Erlenmeyer flasks containing certain amount of adsorbent and 50 mL of aniline solution. The samples then were placed on shaker at a constant speed of 220 rpm. The impact of pH on adsorption aniline was studied in the range of 2–10 with initial concentration of 50 mg/L and contact time of 240 min. The pH of the solutions was adjusted with hydrochloric acid (0.1 M HCl) and sodium hydroxide (0.1 M NaOH). For the study of adsorption kinetic aniline on AC-Fe_3_O_4_ MNPs the effect of contact time was investigated at the period of 400 min with adsorbent dose of 1 g/L and optimum pH and different initial concentrations of aniline in the range of 50–300 mg/L. Influence of adsorbent dose and different initial aniline concentrations to study of adsorption isotherms at range of 0.5-2 g/L and 50–300 mg/L also investigated, respectively. In order to study of temperature effect and determining thermodynamic parameters of aniline adsorption on adsorbent, adsorption experiments was performed at range of 20-50°C in initial aniline concentration 50–300 mg/L under optimum pH, contact time and adsorbent dose. The amount of aniline adsorbed (q_e_, mg/g) onto the adsorbent at each time and efficiency removal (%) of it was calculated through the equation (1) or (2):

(1) $$q_{e}=\frac{C_{0}{-C}_{e}}{w}$$

(2) $$R\left(\%\right)=\frac{C_{0}{-C}_{e}}{C_{0}}\times 100$$

Where C_0_ and C_e_ are the initial and equilibrium (final) concentrations of aniline (mg/L) and *w* is the concentration of AC-Fe_3_O_4_ MNPs (g/L).

### Adsorption isotherms of aniline

Adsorption isotherm model of Langmuir and Freundlich isotherms were employed to describe experimental adsorption data. The Longmuir isotherm is based on assumption of that the adsorption process take place on homogenous adsorbent surfaces with constant energy, whiles Freundlich isotherm assuming that the adsorption process occurs on heterogeneous surfaces with non-uniform distribution of adsorption heat. The linear equations of two isotherms were (3) for Langmuir and (4) Freundlich is:

(3) $$\frac{C_{e}}{q_{e}}=\frac{1}{k_{1}q_{0}}+\frac{1}{q_{0}}C_{e}$$

(4) $${\text{lnq}}_{\;\text{e}}=\text{lnk}{\;}_{F}+\frac{1}{n}\text{lnC}{\;}_{e}$$

Where q_0_ (mg/g) is the solid phase equilibrium concentration of aniline, k_1_ (L/mg) are Langmuir adsorption constant and C_e_ (mg/L) is the equilibrium concentration of the aniline in the liquid phase. k_F_ (mg/g)(L/mg)^1/n^ ) and n (unitless) are the parameters related to the adsorption capacity and intensity of Freundlich, respectively. The values of n indicate the type of adsorption to be favorable (range of 2–10), moderately difficult (in the range of 1–2) or poor adsorption (n < 1). The fundamental properties of the Langmuir isotherm can be explain in terms of dimensionless separation factor R_L_:(R_L_ = 1/(1 + k_L_C_0_)). The factor of R_L_ indicates the type of the isotherm to be unfavorable (R_L_ > 1), favorable (0 < R_L_ > 1), irreversible (R_L_ = 0) and linear adsorption (R_L_ = 1).

### Adsorption kinetics of aniline

In other to analysis sorption kinetic data adsorption process of aniline onto AC-Fe_3_O_4_ MNPs, two kinetic models, pseudo-first-order and pseudo-second-order models, were applied to establish the best fitted model for the experimental data. The linear equation of two models can be expressed as (5) for first-order and (6) second order

(5) $${\text{ln}(q}_{e}{-q}_{t}{)=\text{lnq}}_{e}{-k}_{1}t$$

(6) $$\frac{t}{q_{t}}=\frac{1}{k_{2}q_{e}{}^{2}}+\frac{1}{q_{e}}t$$

Where qe and qt (mg/g) are the amount of aniline adsorbed at equilibrium and at time t (min), respectively. k1 (1/min) and K2 (g/(mg.min)) are rate constants of the first order and second order adsorption, respectively.

### Aniline adsorption thermodynamics

In order to thermodynamic study of the adsorption process, determination of the main three parameters requires. These parameters included: the standard enthalpy (ΔH^o^), the standard free energy (ΔG^o^) and the standard entropy (ΔS^o^). The amounts of ΔH^o^ and ΔS^o^ calculated using from the intercept and slope of vant Hoff plots of lnK_d_ versus 1/T, respectively and the following equation:

(7) $${\text{lnk}}_{c}=-\frac{\text{ΔH}{}^{\circ}}{\text{RT}}+\frac{\text{ΔS}{}^{\circ}}{R}$$

(8) $$k_{c}=\frac{q_{e}}{C_{e}}$$

Where k_c_ (L/g) is the distribution coefficient, q_e_ and C_e_ are the amount of aniline adsorbed at equilibrium (mg/g) and equilibrium concentration in solution (mg/L), respectively. R (8.314 J/mol.K) is universal gas constant and T (°K) is the solution temperature. The values of ΔG^o^ are calculated from the following equation:

(9) $$\Delta G^{o}=-\text{RTIn}k_{c}$$

## Results

**Figure 1:**
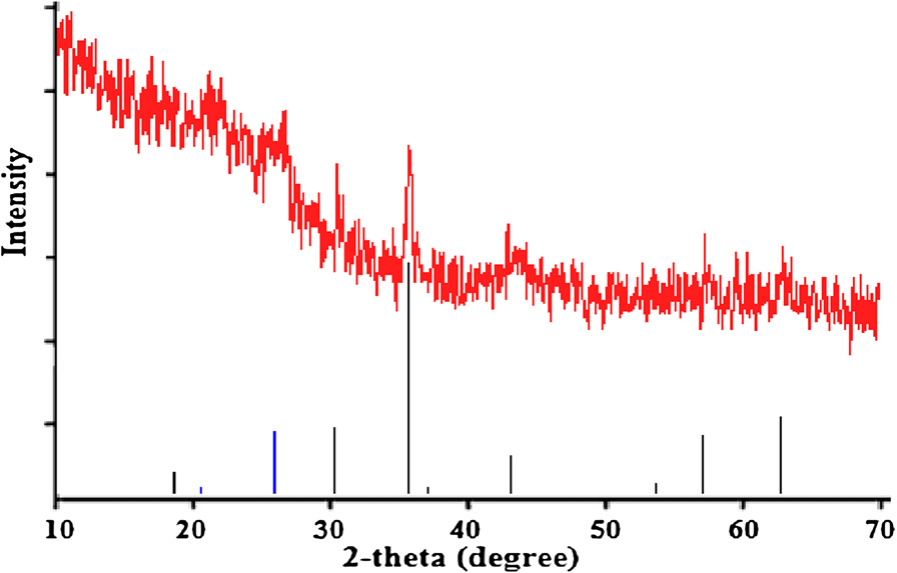
**Spectra of activated carbon-Fe**_**3**_**O**_**4 **_**magnetic nanoparticles.**

**Figure 2:**
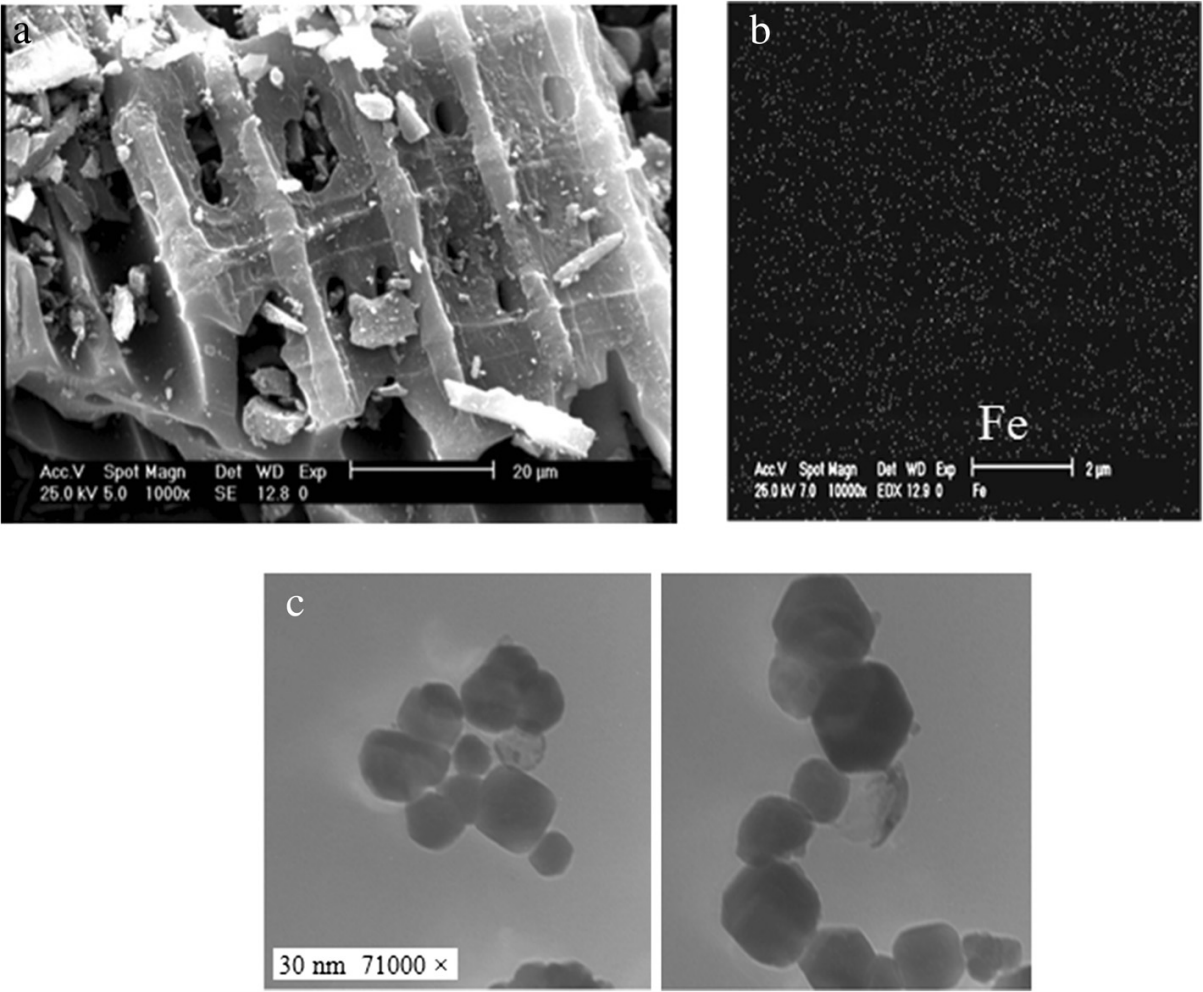
**SEM (a and b) and TEM(c) images of activated carbon-Fe**_**3**_**O**_**4 **_**magnetic nanoparticles.**

**Figure 3:**
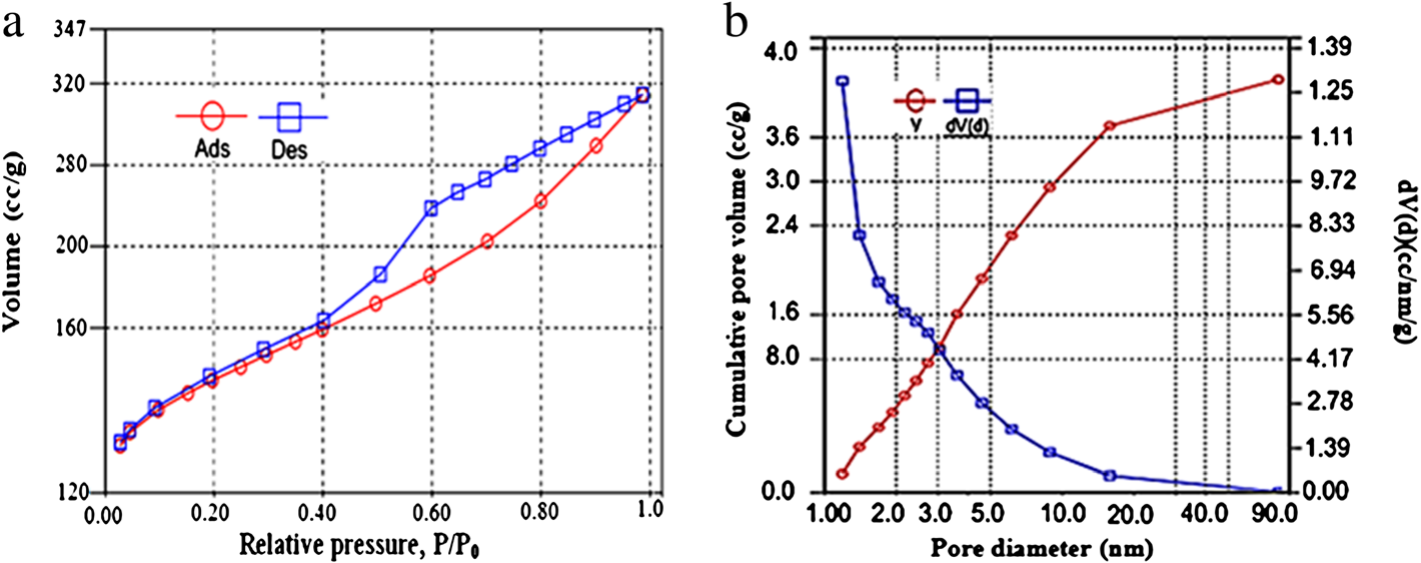
**Nitrogen adsorption/desorption isotherms at 77.3°K (a), pore size distribution of activated carbon-Fe**_**3**_**O**_**4 **_**magnetic nanoparticles (b).**

**Figure 4:**
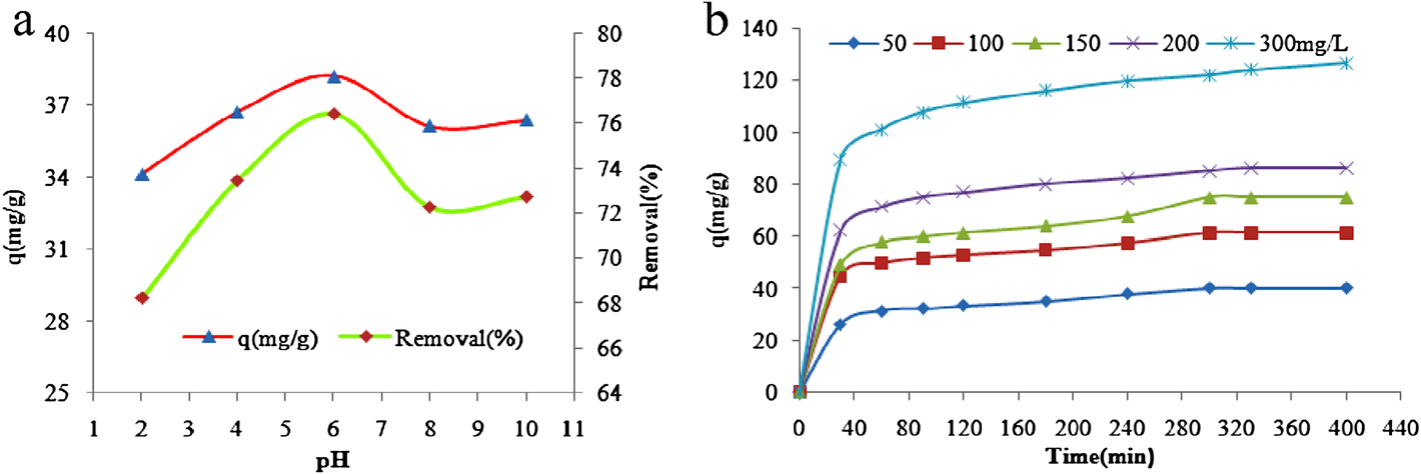
**Effect of pH (a) in C**_**0 **_**= 50 mg/L and contact time (b) in C**_**0 **_**= 50-300 mg/L for aniline adsorption on AC-Fe**_**3**_**O**_**4 **_**magnetic nanoparticles, W = 1 g/L and T = 20°C.**

**Figure 5:**
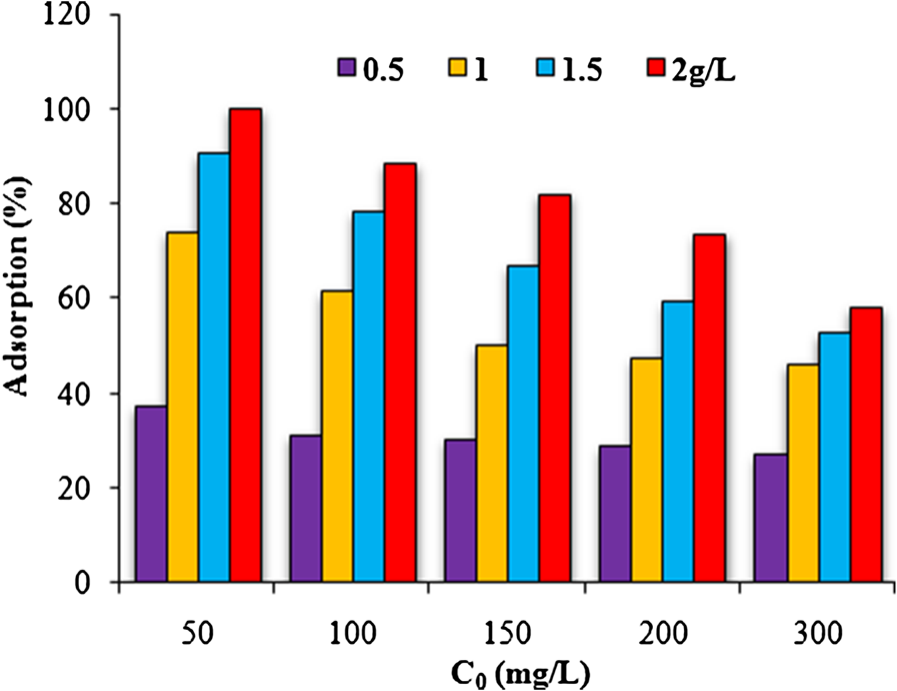
**Effect of various aniline concentrations on its adsorption upon AC-Fe**_**3**_**O**_**4 **_**magnetic nanoparticles: pH = 6, t = 300 min, C**_**0 **_**= 50-300 mg/L W = 0.5-2 g/L and T = 20°C.**

**Figure 6:**
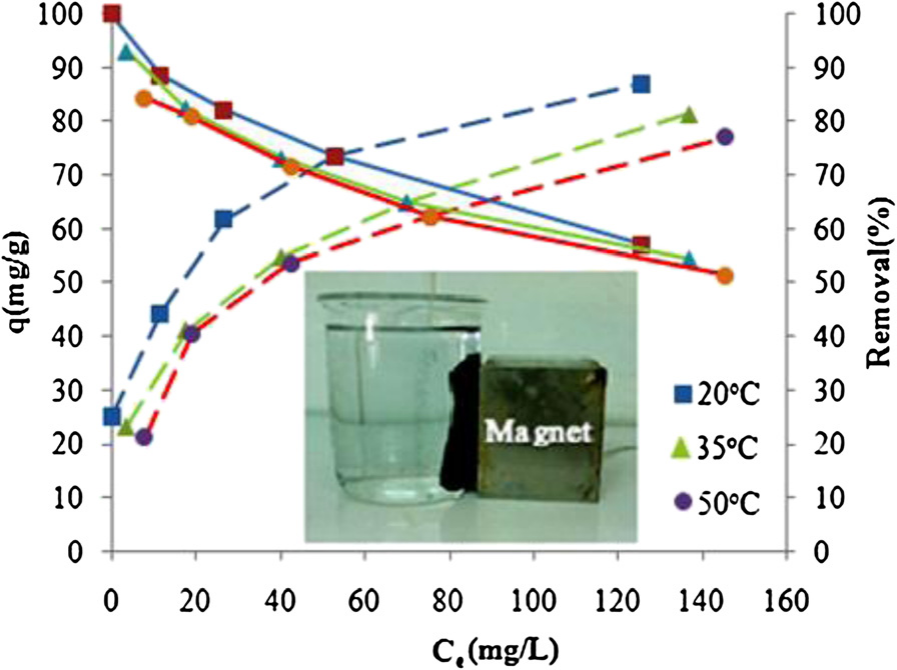
**Impact of various temperatures on aniline adsorption upon AC-Fe**_**3**_**O**_**4 **_**MNP**_**S**_**: pH = 6, t = 300 min, C**_**0 **_**= 50-300 mg/L, W = 2 g/L and T = 20-50°C.**

**Figure 7:**
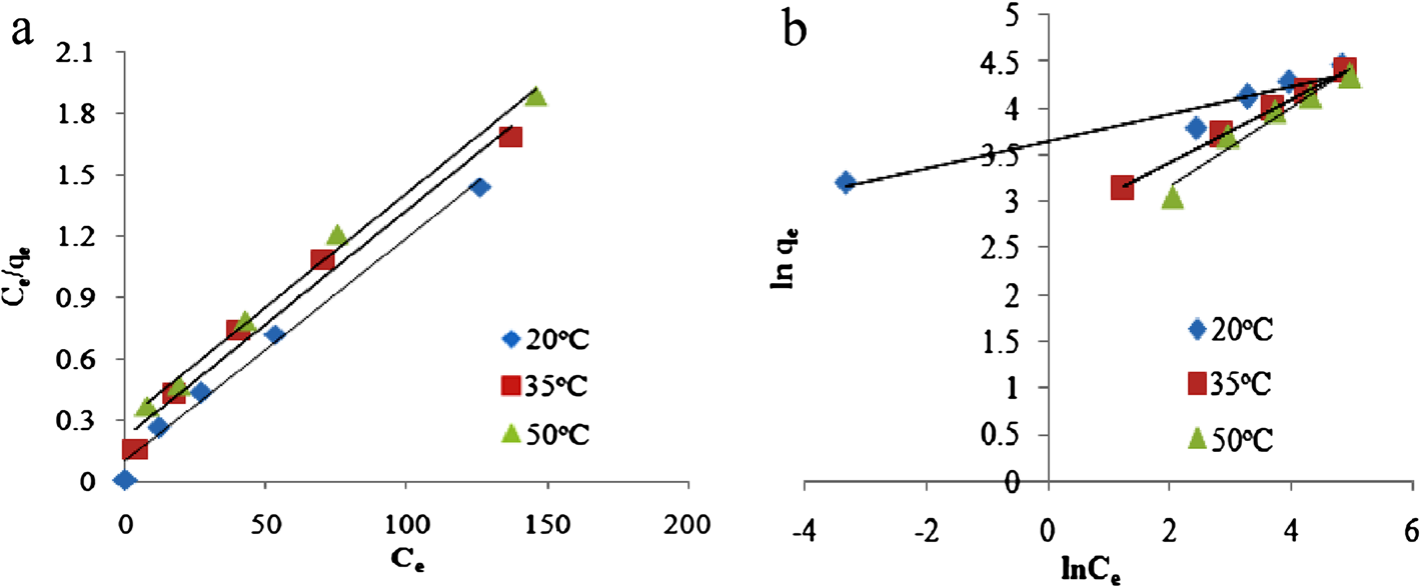
**Langmuir (a) and Freundlich (b) models for aniline adsorption on AC-Fe**_**3**_**O**_**4 **_**MNP**_**S**_**.**

**Figure 8:**
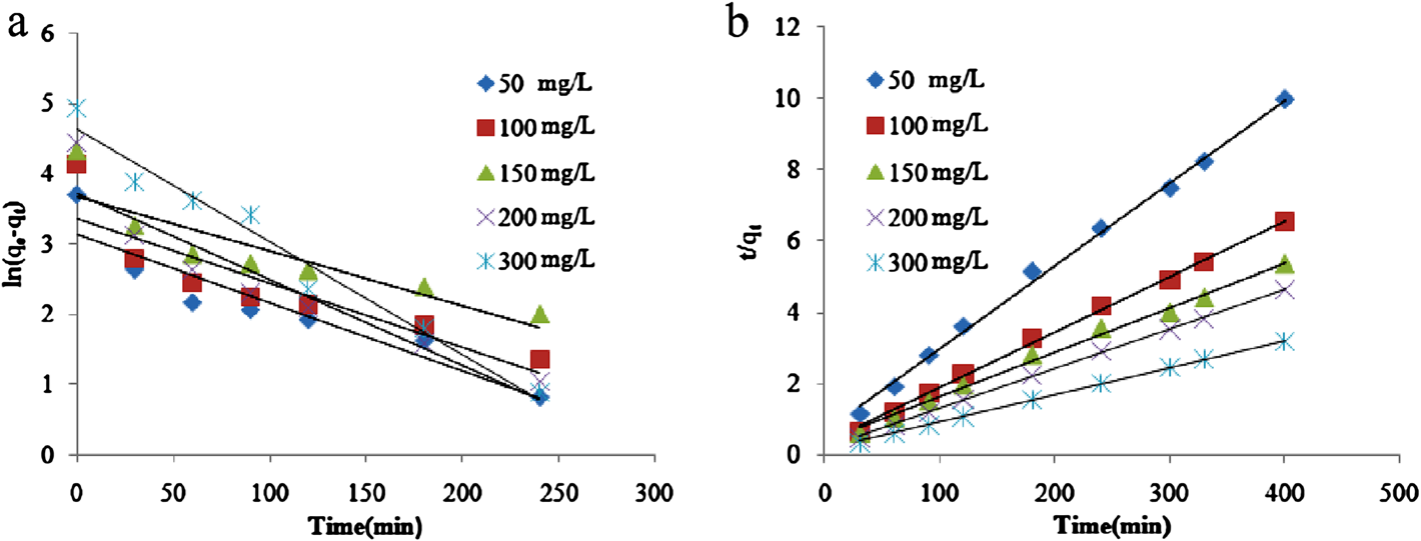
**The kinetic models for aniline adsorption on AC-Fe**_**3**_**O**_**4 **_**MNPs: (a) Pseudo-first order, (b) Pseudo-second order.**

Figures 1, 2 and 3 show the physical and morphology properties of synthesized adsorbent by SEM, TEM, XRD and BET techniques. Effect of various pH = 2-10 in adsorption efficiency of aniline on AC-Fe_3_O_4_ MNPs in 4 hours contact times is shown in Figure 4(a). This figure shows that with increasing pH from 2 to 6 the adsorption efficiency increased and then decreased in higher pH. Maximum aniline adsorption performance was in pH = 6 (76.4%) that the adsorption capacity was 38.21 mg/g and lowest amount of adsorbed aniline (34.1 mg/g) related to pH = 2, and the removal efficiency was 68.3%. Figure 4(b) shows the influence of contact time for aniline adsorption onto synthesized adsorbent in different concentrations of aniline (50–300 mg/L) in the optimum pH. The effect of different concentrations of adsorbent and adsorbate in pH and optimal contact time on adsorption efficiency has been observed in Figures 5 and 6 shows the impact of different temperatures on the adsorption process of aniline in optimized pH, contact time, adsorbent dose and various aniline concentrations (50–300 mg/L). As shown in Figure 6 with the increasing of temperature from 20 to 50°C in initial concentrations of 50, 100, 150, 200 and 300 mg/L of aniline, the efficiency decreased from 99.9, 88.5, 82.2, 73.5 and 57.07% to 84.4, 80.8, 71.6, 62.3 and 51.4%, respectively. Also, the adsorption capacity decreased from 25, 44.3, 61.7, 73.5 and 87.1 mg/g to 21.1, 40.4, 53.7, 62.3 and 77.2 mg/g, respectively. The Langmuir and Freundlich isotherms linear plots are shown in Figure 7. The parameters related to Langmuir and Freundlich isotherm models are presented in Table 1. The maximum adsorption capacities and Langmuir constant in the Langmuir model at different temperatures of 20, 35 and 50°C were 90.91, 90.1 and 91.74 mg/g, and 0.115، 0.05 and 0.035 L/mg, respectively. The parameters values obtained from adsorption process kinetics AC-Fe_3_O_4_ MNPs have been showed in Table 2. Figure 8 shows the adsorption kinetics curves for aniline. The parameters values of aniline adsorption thermodynamic on magnetic nanoparticles of AC-Fe_3_O_4_ are shown in Table 3.

**Table 1:** **Parameters of adsorption equilibrium isotherms for An on AC-Fe**_**3**_**O**_**4 **_**MNPs**

**Isotherm models**	**T(°K)**		
	**293**	**308**	**323**
**Lungmuir**			
q_m_(mg/g)	**90.91**	**90.1**	**89.285**
K _L_(L/mg)	**0.115**	**0.05**	**0.037**
R^2^	**0.9876**	**0.981**	**0.994**
R_L_	**0.028-0.148**	**0.062-0.285**	**0.082-0.351**
**Freundlich**			
K_F_ (mg/g(Lmg)^1/n^)	**38.424**	**15.27**	**9.81**
1/n	**0.1474**	**0.342**	**0.4328**
R^2^	**0.9317**	**0.9995**	**0.955**

**Table 2:** **Kinetic parameters for adsorption of An on AC-Fe**_**3**_**O**_**4 **_**MNPs**

		**Pseudo-first order**			**Pseudo-second order**		
**Initial an concentration**	**q**_**e,exp**_	**q**_**e,cal**_**(mg/g)**	**K**_**1**_**(1/min)**	**R**^**2**^	**q**_**e,cal **_**(mg/g)**	**k**_**2**_**(g/(mg.min))**	**R**^**2**^
**50**	40.14	23.021	0.0098	0.8718	42.918	0.0008	0.997
**100**	61.293	28.849	0.0092	0.7929	64.102	0.0007	0.9977
**150**	74.93	39.377	0.0078	0.784	80	0.0004	0.9942
**200**	85.07	41.227	0.0123	0.8783	90.09	0.0006	0.9994
**300**	122.0	102.74	0.016	0.969	129.870	0.0004	0.9993

**Table 3:** **Thermodynamic parameters of aniline adsorption on AC-Fe**_**3**_**O**_**4 **_**MNPs**

**Temperature (°K)**	**lnk**_**c**_	**∆ G**^**0**^**(kJ/mol)**	**∆ H**^**0 **^**(kJ/mol)**	**∆ S**^**0 **^**(kJ/mol.K)**
**298**	6.542	−16.208		
**308**	1.893	−4.847	−147	−0.452
**323**	0.994	−2.67		

## Discussion

### Characterization of the adsorbent

In order to determine crystal phase of iron oxide particles, the sample were analyzed by powder X-ray diffraction (XRD) in the 2θ in region of 10-70^o^ at 25°C by using Cu kα radiation (λ = 1.54Å). Figure 1 shows XRD pattern for the synthesized adsorbent. The highest peaks of AC-Fe_3_O_4_ MNPs at 2θ value of 30.2^o^، 35.5^o^، 54.9^o^ and 62.9^o^ corresponded to (172), (342), (511), (122) and (106) planes, which by comparison with JCPDS NO. 01-088-0866 was confirmed presence crystals of magnetic nanoparticles Fe_3_O_4_ with a cubic structure (2θ = 35.5^o^). So, this analysis illustrate that Fe_3_O_4_ magnetic nanoparticles were on powder activated carbon successfully were synthesized. In previously studies similar this results was reported [18,19].

The morphology, porosity and texture structure of samples evaluated by using scanning electron microscopy (SEM, Philips XL30) at 25 keV (Figure 2). It can be seen porous with different sizes and shapes and indicated that their distribution on surface of the adsorbent was nearly uniform (Figure 2(a)). Figure 2(b) shows the dispersion of Fe element on activated carbon and so, suggested that nanoparticles iron oxide (Fe_3_O_4_) on the adsorbent was alike. In order to investigate size and shape of Fe_3_O_4_ nanoparticles, sample was analyzed by using TEM micrographs at 100 keV (Figure 2c), it shows the iron oxide particles with average diameter of 30-80 nm. Figure 2(c) also shows Fe_3_O_4_ nanoparticles with a cubic structure which is compatible with results obtained from the analysis of XRD.

Figure 3 shows the N_2_ adsorption and desorption isotherms, volume and pore size distribution for magnetic nanoparticles of AC-Fe_3_O_4_ by applying the methods of BET and BJH at 77.3°K. According to classification of IUPAC, Figure 3(a) exhibit type 4 adsorption isotherms, that reveals the presence of mesopores in adsorbent. Similar results were obtained by another researcher [20]. The results of the BET analysis indicated that the highest surface area of adsorbent was 671.2 m^2^/g which, in comparison with non-magnetic PAC (1301 m^2^/g) was reduced [21]. It can be due to presence of Fe_3_O_4_ nanoparticles in the structure of activated carbon [16,21]. Faulconer and et.al reported available surface area decreasing by iron oxides increase, For example at relative of 1:1 C:Fe surface area decreased approximately a 50% [22]. The average pore size measured by BET and BJH was 3.5 and 1.2 nm, respectively which based on IUPAC classification (micropores (d < 2 nm), mesopores (2 < d < 50 nm) and macropores (d > 50 nm)), it (mean size of 3.5 nm) could be classified in mesopores group and the total pore volume by BET obtained 4.87 cc/g at p/p_0_ = 0.99 and by BJH was 3.7 cc/g (Figure 3(b)).

### Optimal conditions for aniline adsorption on magnetic nanoparticles AC- Fe_3_O_4_

#### Optimal pH for uptake of aniline

According to Figure 4(a) in very low pH, electrostatic repulsion between the positive protons of the surface of activated carbon and positive molecules of aniline (or intense competition between H^+^ and positive charged molecules of aniline) lead to decrease of adsorption capacity. On the other hand, in alkaline pHs, the repulsion of negative charges on the adsorbent and aniline will reduce the adsorption of aniline. The decrease of aniline adsorption under alkaline pH condition may be due to the presence of excess OH^-^ ions and the aniline molecules for the adsorption sites [23,24]. Zhang and et al. reported the acidic and neutral conditions for favorable removal of aniline using nanoparticles of Fe_3_O_4_[25]. Tang et al. reported pH = 6.5 as optimal pH for removal of aniline with granular activated carbon [24]. Therefore pH 6 selected as optimal pH in removing of aniline on magnetic nanoparticles of AC-Fe_3_O_4_ and next experiments were conducted in this pH.

#### Optimal contact time for aniline adsorption

Figure 4(b) reveals that with increase of time from 0 to 400 min was increased the absorbed aniline and it reached to equilibrium in 5 h for the concentrations of 50, 100 and 150 mg/L of aniline and then complete absorption because absorptive capacity remained constant. But in initial concentrations 200 and 300 mg/L the time to reach equilibrium became longer. This may be due to large number of aniline molecules and the dispersion rose from their impact to boundary layer of absorbent. So, the optimal contact time was selected 300 min for absorption of aniline using magnetic nanoparticles. This contact time for removal of aniline by An and et al. reported [26]. Adsorption efficiency at this time for initial concentrations of 50, 100, 150, 200 and 300 mg/L were 80.3, 61.3, 49.9, 42.5 and 40.7%, respectively.

#### Effect of initial aniline concentration and adsorbent dose

Fig**ure**5 demonstrates that by increasing the initial concentration of aniline from 50 mg/L to 300 mg/L in 2 g/L of adsorbent dose, the efficiency decreased from 100 to 58%. The increasing of adsorption efficient in this case may be attributed to the fact that all the adsorbents has a limited number of active sites, which would has become saturated at a specific concentration [27]. However vice versa, adsorption capacity increased with increasing initial aniline concentration. This can be explained that driving forces rising from increasing the aniline concentration [28]. Figure 5 also shows that with increase of adsorbent dose from 0.5 to 2 g/L, in initial concentration of 50 mg/L, enhances the removal efficiency from 37.2 to 100% due to increase sorbent surface or active sites. The increase the in the adsorption efficiency can be due to the increasing availability of aniline molecules to adsorbent surface [29]. The similar results were reported in elimination of aniline with Fe_3_O_4_ nanoparticles and activated carbon obtained from oxygen plasma irradiated bamboo [4,25]. Hence in present study, the amounts of 2 g/L were chosen as optimal adsorbent doses.

#### Influence of solution temperature on aniline adsorption

As displayed in Figure 6 the results show that with increasing temperature both removal percent and adsorption capacity reduced. Al-Johani et al. (2011) and Tang et al. (2012) in study of aniline removal by carbon nanotube and activated carbon were reported decrease of adsorption efficiency with increasing of temperature [24,30]. Therefore, decreasing the removal efficiency with increasing temperature indicates that aniline adsorption on AC-Fe_3_O_4_ magnetic nanoparticles is exothermic.

#### Adsorption isotherms

It is clear from Table 1 that the correlation coefficient in Longmuir model, for all three investigated temperatures was higher than 0.98. It also observed that the values of R_L_ are between 0 and 1. This shows that the aniline molecules are desirably adsorbed on adsorbent. Thus, according to the regression coefficients obtained from Langmuir and Freundlich models, the adsorption process of aniline follows better from Langmuir model. This suggests that the aniline adsorption on AC-Fe_3_O_4_ MNPs was monolayer. Similar results have been reported by Tang et.al (2012) and Wuet.al (2012) in aniline removal using activated carbon [4,24].

#### Adsorption kinetics

Table 2 shows that the regression coefficients in pseudo second order kinetic model of all initial studied concentrations in compare with the pseudo first order model were higher or almost equal to unit. It also shows that the amounts of calculated capacity (q_e,cal_) in the pseudo second order model rather than the pseudo first order model were more closer and consistent with adsorption capacity obtained from experiments(q_e,exp_). According to previous studies, this result suggests that the adsorption behavior of aniline by the studied adsorbent per time unit follows from pseudo second order model and it indicates that the rate-limiting step in aniline adsorption process may be chemisorptions [30,31].

#### Aniline adsorption thermodynamic

As shown in Table 3 the values of ΔH^o^ are negative and also ΔG^o^ values for all three temperatures 20, 35 and 50°C were positive. The negative amounts of ΔH^o^ and ΔG^o^ show that the adsorption process of aniline on AC-Fe_3_O_4_ MNPs is exothermic and spontaneous. Decreasing of ΔG^o^ with increasing temperature indicates that the adsorption process at higher temperature is undesirable. The negative amounts of ΔS^o^ also indicated that the efficiency is reduced with increasing temperature in solid /liquid phases during adsorption process (or degree of entropy decreases in during the adsorption process). This phenomenon can be resulted from minor changes in the structure of adsorbate and adsorbent at different temperatures.

## Conclusions

The magnetic nanoparticles of AC-Fe_3_O_4_ with combining iron oxide nanoparticles (IONPs) and powder activated carbon, were successfully synthesized and used in the removal of aniline from aqueous environments. Results revealed that the adsorption efficiency of aniline on this adsorbent was better at pH close to neutral and it was enhanced with increasing the contact time and adsorbent dosage, but reduced with increasing initial aniline concentration and temperature. Equilibrium and kinetic studies indicated that the adsorption was fit with Langmuir and pseudo second order, respectively and thermodynamic studies also showed that the adsorption aniline on AC-Fe_3_O_4_ MNPs has been spontaneous and exothermic. The present study showed that nanoparticles of AC-Fe_3_O_4_ in addition to having features such as easy separation and extraction solution and need no filtration, have proper porosity, surface area and also good adsorption capacity. This adsorbent can be used as effectively and efficiently adsorbent to remove most contaminants, especially organic pollutants from aquatic environment.

## Competing interests

The authors declare that they have no competing interests.

## Authors’ contribution

BK carried out all the experiments of the work and also wrote the whole article. AJJ, RRK give Idea of research and the research was done under their supervision and read, SN and AA read and corrected the manuscript. AJJ read and finalize the manuscript. AE helped with all the experiments presented in this paper. All authors read and approved the final manuscript.
